# Virtual day center for people with dementia and their caregivers during the COVID-19 pandemic

**DOI:** 10.1590/1980-57642021dn15-040003

**Published:** 2021

**Authors:** Virgínia Lúcia Reis Maffioletti, Maria Alice Tourinho Baptista, Bethânia Abranches, Gabriela Koatz, Valeska Marinho Rodrigues, Andrea Deslandes, Marcia Cristina Nascimento Dourado

**Affiliations:** 1 Center for Alzheimer’s Disease, Institute of Psychiatry, Universidade Federal do Rio de Janeiro – Rio de Janeiro, RJ, Brazil.

**Keywords:** adult day care centers, dementia, pandemic, caregivers, centros-dia de assistência à saúde para adultos, demência, pandemia, cuidadores

## Abstract

The coronavirus disease 2019 (COVID-19) pandemic suspended face-to-face assistance offered by community spaces, such as day centers (DCs). People with dementia (PwD) and their families were faced with the risks posed by social detachment and suspension of treatments.

**Objective:**

This study aimed to present a virtual day center (VDC) program offered as a preventive strategy to reduce the damage caused by social isolation and interruption of treatment imposed by the pandemic.

**Methods:**

The experience report, describing the feasibility of a VDC program, offered to 26 PwD and their caregivers, during the first year of the pandemic. The VDC held individual and group meetings with PwD and their families and psychoeducational support groups for caregivers.

**Results:**

The attendance rate in group activities was 80%, and that in the caregiver group was 68%, showing a good virtual interaction. Throughout the year, three PwD interrupted the service due to difficulties of the caregivers to reconcile the schedules of the activities with their professional commitments and the absence of a support network, three others died, and two were institutionalized. PwD remained physically, socially, and cognitively active through daily virtual activities. Guidance and support for caregivers contributed to the organization of routines and adaptation to the isolation and maintenance of the bond. Family meetings made it possible to mediate conflicts and expand the support network.

**Conclusions:**

VDC is a promising modality to assist the needs and demands of PwD and their caregivers. VDC can contribute to the expansion of this intervention to individuals and families who do not have access to face-to-face treatment.

## RESUMO

A pandemia da COVID-19 impôs a suspensão da assistência presencial oferecida por espaços comunitários, como centros dias. Pessoas com demência (PcD) e seus familiares enfrentam os riscos do distanciamento social e da suspensão dos tratamentos.

**Objetivos:**

Apresentar um programa de centro dia virtual (CDV) oferecido como estratégia preventiva para reduzir os danos causados pelo isolamento social e interrupção do tratamento impostos pela pandemia.

**Métodos:**

Relato de experiência, descrevendo a viabilidade de um programa VDC, oferecido a 26 PcD e seus cuidadores, durante o primeiro ano da pandemia. O CDV realizou reuniões individuais e em grupo com PcD e seus familiares e grupos de apoio psicoeducacional para cuidadores.

**Resultados:**

A taxa de comparecimento nas atividades em grupo foi de 80% e nos grupos de cuidadores foi 68%, demonstrando uma boa interação virtual. Ao longo do ano, três PcD interromperam o atendimento por dificuldade dos cuidadores em conciliar os horários das atividades com seus compromissos profissionais e ausência de rede de apoio, outros três faleceram e dois foram institucionalizados. PcD mantiveram-se física, social e cognitivamente ativas por meio de atividades virtuais diárias. A orientação e o apoio aos cuidadores contribuíram para a organização das rotinas, a adaptação ao isolamento e manutenção do vínculo. As reuniões familiares possibilitaram mediar conflitos e ampliar a rede de apoio.

**Conclusões:**

o CDV é uma modalidade promissora para atender às necessidades e demandas de PcD e seus cuidadores. O CDV pode contribuir para a expansão dessa intervenção a indivíduos e famílias que não têm acesso ao tratamento presencial.

## INTRODUCTION

The coronavirus disease 2019 (COVID-19) pandemic has burdened health systems.^1,2,3,4^ The emergency preventive action was social distancing, with circulation authorized only for basic needs.^3,4^ To reduce its traumatic impact on individuals, families, and communities, it requires agility of the social responsibility in the organization of prevention and intervention strategies.^1,2^

The vulnerability of the elderly, such as hypertension, diabetes^1^ has been correlated with increased morbidity in COVID-19,^5^ and people with dementia (PwD) have an increased risk of COVID-19.^6^ In addition, PwD would have a higher risk of contamination due to cognitive impairment, which would limit their ability to understand the pandemic and the need for biosafety procedures, such as washing hands, wearing masks, and maintaining social distance.^7^ COVID-19 can worsen cognitive impairment and increase the incidence of psychological and behavioral changes, temporarily during the infectious process, or permanently if the duration of the infectious condition is very long.^1^

Social detachment can increase the feeling of loneliness, emotional isolation, and disconnection from existing relationships,^8,9,10,11,12^ aggravating the situation of socioemotional isolation frequently reported by PwD caregivers.^1,2,13,14,15,16^ Livingston et al.^8^ suggested that social isolation is a risk factor for dementia, presenting a relative risk higher than the risk associated with alcohol abuse, physical inactivity, diabetes, and air pollution.

The pandemic suspended outpatient care and the closure of community and psychosocial care spaces, such as the day centers (DCs). Consequently, the PwD and their families began to experience a critical situation, given the risk and impact of discontinuing treatment.^1^ This context imposed the reorganization and flexibility of families, health services, and community support networks.^1,2^ For these reasons, the team of the DC for PwD of the Center for Alzheimer’s Disease and Other Mental Disorders in Old Age (CDA) of the Psychiatry Institute (IPUB) of the Federal University of Rio de Janeiro (UFRJ) restructured the intervention to a model totally virtual. This experience report presents a VDC for PwD and caregivers, from March 2020 to February 2021, during the COVID-19 pandemic.

## METHODS

We designed a qualitative study to report a virtual day center (VDC) experience offered for 26 PwD and their caregivers. This study is part of the research project approved by the Ethics Committee of IPUB/UFRJ (CAAE: 80423217.4. 0000.5263). All PwD and their caregivers signed the informed consent form. Demographic and clinical data, frequency of activities, and information on PwD and caregivers were collected from medical records. The availability of Internet resources, computers, and tablets was informed through the telephonic interviews.

### Statistical analysis

Descriptive statistics were used to present the sociodemographic and clinical data of PwD, which were assessed through in the medical records by the attending physician at the last face-to-face consultation, gender, education, marital status, diagnosis, age, Clinical Dementia Rating (CDR),^17^ Mini-Mental State Examination (MMSE),^18^ duration of the disease, treatment duration, and caregivers (type of bond, gender, cohabitation and presence of formal caregiver). The descriptive data analysis was performed using SPSS® software, version 26.0 (IBM Corporation, Endicott, NY, USA).

## RESULTS

Most of the PwD (80.76%) are at the age of 70–89 years and have a diagnosis of Alzheimer’s disease (AD; 80.8%). According to the CDR,^17^ 34.6% were in the mild stage with a mean MMSE^18^ score of 24.4, 38.5% were in the moderate stage with a mean MMSE score of 19.1, and 26.9% were in the severe stage with a mean MMSE score of 8.5. The mean attendance time was 61.6 months, with a range of 14–189 months. The sociodemographic and clinical data are shown in Table 1.

**Table 1.** Characteristics of people with dementia and their family caregivers.

People with dementia						
Gender	n					(%)
Male	11					(42.3)
Female	15					(57.7)
Marital status						
Single	1					(3.8)
Married	9					(34.6)
Widower	12					(46.2)
Divorced	4					(15.4)
Diagnosis						
Alzheimer’s disease	16					(61.5)
Vascular dementia	4					(15.4)
Others	6					(23.1)
Schooling						
0–4 years	6					(23.1)
5–8 years	6					(23.1)
9–12 years	6					(23.1)
>12 years	8					(30.8)
CDR						
1	9					(34.6)
2	10					(38.5)
3	7					(26.9)
Age (years old)						
60–69	2					(7.7)
70–79	8					(30.8)
80–89	13					(50.0)
≥90	3					(11.5)
Treatment status						
In treatment	18					(69.2)
Dead	3					(11.5)
Institucionalized	2					(7.7)
Broke off	3					(11.5)

Attendance	Frequency — n					Attendance rate (%)
Individual assistance	658					(100)
Group meetings	962					(80)
Family meetings	33					(100)
Caregiver groups	529					(68)

Age (years old)	CDR			Mean illness time		
	1(%)	2(%)	3(%)		SD	Min-Max
60–69	1(3.8)		1(3.8)	8.0 years	4.2	5–11
70–79	5(19.2)	3(11.5)		6.1 years	5.9	1–15
80–89	3(11.5)	6(23.1)	4(15.4)	7.7 years	3.8	2–15
≥90		1(3.8)	2(7.7)	13.6 years	8.3	4–19
Mean MMSE 2019	24.4 (21–28)	19.1 (12–24)	8.5 (6–13)			
Mean attendance time on the DC	61.1 months (SD 45.2, 14–189)					

CDR: Clinical Dementia Rating; MMSE: Mini-Mental State Examination; DC: day center.

The PwD showed satisfaction during the virtual meetings. The majority of PwD and their caregivers (69%) participated in the activities and groups of caregivers during the 12 months of the program. Three PwD discontinued after 3 (8%) and 6 months (4%), respectively. Two were institutionalized (8%) after 7 and 9 months of care, respectively, and three died after 2 (4%) and 9 (8%) months of participation, respectively.

The majority of PwD (76.9%) cohabited with their caregivers, being cared for their children (65.4%) or spouses (30.8%). Most of the caregivers (96.2%) were females, and 23.1% did not have a family support network. All of them had access to a smartphone with Internet access, and approximately 30% had a computer or tablet. The sociodemographic data of the caregivers are shown in Table 2.

**Table 2.** Characteristics of family caregivers.

Caregivers	n	(%)
Gender		
Male	1	(3.8)
Female	25	(96.2)
Schooling		
Until 8 years	2	(7.7)
Until 12 years	3	(11.5)
>12 years	21	(80.8)
Age (years)		
<45	4	(15.4)
45–65	15	(57.7)
66–75	5	(19.2)
>75	2	(7.7)
Kinship		
Spouse	8	(30.8)
Children	17	(65.4)
Others	1	(3.8)
Cohabitation		
Yes	20	(76.9)
No	6	(23.1)
Formal caregiver		
Yes	14	(53.8)
No	12	(46.2)
Support network		
Family	10	(38.5)
Only formal caregiver	4	(15.4)
Family+formal	10	(38.5)
None	2	(7.7)

It was observed in the caregiver’s organization of routines, adoption of creative mediation strategies in the care relationship, and new stress-mediating practices, such as meditation and aromatherapy. Caregivers shared their experiences with peers in groups of caregivers with a frequency rate of 68%. Most of the families (54%) who participated in family meetings, which contributed to the engagement of new members of the family network in care, expanded the support network of caregivers.

### The Day Center

The DC offers an intervention program that assists PwD referred by the clinical staff of the CDA outpatient clinic after diagnosis. Before the pandemic, users attended the service accompanied by a caregiver, twice a week, staying 4–6 hours a day. The team includes psychologists, occupational therapists, music therapists, and students from the Specialization Course in Psychogeriatrics. The therapeutic program includes cognitive, motor, and psychosocial stimulation activities, which is preferably carried out in groups, i.e., psychoeducation groups, and support for caregivers, food, and recreational tours.^19^ The DC is theoretically guided by the Reuven Feuerstein’s theory of Mediated Learning Experience, whose paradigms are the structural cognitive stimulation, the centrality of the caregiver role, and the modification of the environment.^20,21,22^ In addition, the Conceptual Model of Alzheimer’s Caregivers Stress proposed by Pearlin et al.,^13,14^ which considers the impact of primary and secondary stressors on caregiver burden.

### The Virtual Day Center

The implantation of the VDC was performed in the following three steps.

#### STEP 1

In the second half of March 2020, the DC staff established an emergency intervention plan with the objective of minimizing the risks and mitigating the suffering of its users. The first virtual strategy was to establish contact by video call, twice a week, with the caregivers and PwD who attended the DC. The 26 PwD were divided into three groups, considering the level of cognitive and functional impairment. Each group was accompanied by a team member, two psychologists, and an occupational therapist, respectively. The initial objectives were to maintain the link with PwD and their family; know how families were organizing, their understanding of the pandemic and biosafety measures; identify risk and protection factors and reinforce coping strategies; identify patterns of dysfunctional responses and mediate the adoption of new behaviors; and advise on low-risk alternatives for the purchase of food and medicines and what procedures should be adopted in case of contamination of the PwD or caregiver.

To facilitate the development of a repertoire of more functional and less impulsive behavioral strategies, the team implemented two actions as follows:

- Four videos were recorded and sent via WhatsApp, addressing topics such as the pandemic, its risks, psychological impact, and coping and biosafety strategies; the organization of routines; and virtual strategies for socio-affective interaction and recreational activities. The Physical Activity and Psychoeducation Program for Autonomous Life with Quality (PROAPTIVA), a CDA extension project, recorded four videos with movement guidelines to encourage mobility and avoid a sedentary lifestyle.
- Development of nine handouts with cognitive stimulation activities, with different levels of complexity to meet the PwD in their different commitment profiles. The handouts were sent via email or post.

At this stage, 100% of caregivers adhered, and the use of technology did not represent an obstacle. In some cases, we observed difficulties in understanding the need for biosafety rules and restricting visits, family conflicts, breaking contracts with formal caregivers, and overlapping tasks for caregivers. Some caregivers showed risk behaviors and anxious responses, such as agitation, unnecessary exits, difficulty sleeping, obsessive cleaning, irritability, and need for exacerbated control in the management, infantilization, and demands incompatible with the cognitive and functional limitations of PwD. In these cases, the caregivers reported and the professionals observed during the video calls that some PwD had disorientation, agitation, apathy, depression, and daytime sleepiness. The active listening offered in video calls helped the caregivers to express feelings and reflect on the patterns of behavior adopted, in addition to assisting in the adoption of more adaptive behaviors. In contrast, some caregivers showed greater resilience and adaptive coping strategies that favored family life.

For the development of the work, the team held weekly supervision meetings, in which the cases were discussed and the activities planned. These meetings also served to welcome team members into their own fears and difficulties in dealing with the pandemic.

#### STEP 2

From the first week of April, the focus was to help PwD and caregivers to establish a routine that would organize the experience of seclusion at home, encouraging self-care practices and socio-interactive activities. The PwD and their caregivers participated in a video call per week, lasting 60 minutes, comprised of 2 dyads. The pairs of PwD were formed considering their cognitive profiles. We started our first experience of a virtual group activity with 100% of user adhesion. In the meetings, we encouraged the exchange of experiences between PwD and caregivers. We talked about biosafety care and carried out the activities of cognitive stimulation (e.g., fluency exercises, calculations, and interpretation of proverbs), by keeping the meeting as playful as possible. We maintained a second individual weekly contact for each dyad, in which we reviewed the exercises of the handouts carried out throughout the week and received individualized demands. Thus, virtual activities, homework, and physical activity guided by the PROAPTIVA videos enabled a more active routine for PwD. The group activity represented a stimulus to sociability, being expected by everyone. For dyads with severe PwD, two individual weekly video calls were added, since virtual interaction was more difficult and required more individualized management. All meetings lasted, on average, for 60-minute sessions.

Regarding technology, cell phone, or computer, some older caregivers were initially reticent. These caregivers as well as mild PwD received training to handle the cell phone (i.e., answer calls, turn on and off microphones and cameras, place the device, and understand Internet interference). The mild PwD achieved greater autonomy in relation to caregivers. In turn, PwD with moderate and severe difficulties needed to be accompanied by caregivers in activities, due to the limitations imposed by sensory difficulties — auditory or visual — and/or cognitive.

After a month of the pandemic, there was no forecast of returning to normal, which required a reconfiguration of the program.

#### STEP 3

The team, PwD, and caregivers were already adapted to virtual activities. Thus, we proposed to start cognitive stimulation groups with four dyads. The groups had 60-minute sessions, twice a week, coordinated by two professionals from the DC team. In response to caregivers’ requests, psychoeducation and support groups for caregivers were initiated weekly. The PROAPTIVA team started to offer virtual groups of physical activity. The training consisted of a supervised program of 45 minutes of multimodal physical exercises (e.g., aerobic, strength, balance, and flexibility) twice a week. The daily virtual activities helped caregivers to implement routines for waking hours, food, hygiene, and sleep. For PwD with severe disabilities, in addition to psychoeducation and cognitive stimulation, individual video calls were added for the practice of physical activity given by a physical educator. Some PwD and caregivers regularly participate in the virtual choir. PwD who were most anxious started in person or group psychotherapy.

Throughout the year, demands for family meetings for various purposes emerged: guidance on biosafety behaviors and on the risks of family visits; activation of family support networks as a socio-affective or operational resource for purchases; and mediation of past conflicts or those that arose during quarantine. Surprisingly, more family members attended the virtual meetings than those that used to happen in person before the pandemic when it was more difficult to reconcile schedules.

The VDC therapeutic program, with regular activities four times a week, will continue until the face-to-face service is authorized. In these 12 months, we included some virtual parties that brought together all users and the team, such as Easter, Christmas, and birthdays. In addition, some resources to stimulate self-care and stress mediation were presented, such as meditation exercises at the beginning of the groups of caregivers, lectures on nutrition, and a minicourse on the use of aromatherapy in daily life.

In the third month of the pandemic, 1 PwD and 1 spouse were diagnosed with COVID-19 and died. At this moment, the activities were permeated by sadness and mourning shared by all participants. In groups, the caregivers reported the fear of death of PwD and themselves. Three PwD interrupted the intervention due to caregivers’ difficulty in reconciling the activities of the VDC with their professional commitments and the absence of a support network. In November, two PwD died but were not due to COVID-19. Two PwD with greater impairment were institutionalized due to their caregiver’s illness. In both cases, institutionalization proved to be the best alternative for other family members. The team analyzed, with these families, the costs and benefits and strategies for adapting PwD to their new environments, given the restriction of visits imposed by the pandemic. In May 2020 and February 2021, we paid two virtual tributes to our deceased friends because the pandemic made the farewell rites fundamental to facilitate the mourning.

During this period, the team’s cohesion was fundamental. The weekly supervision meetings become enriching learning spaces with the revision of the guiding theories in light of new challenges. The adequacy of the intervention methodology took into account the difficulties imposed by technology and the Internet, as well as the specificities of each PwD and their caregiver in their cognitive and socio-affective processes.

## DISCUSSION

The social distance imposed a sudden change in everyone’s routine, mobilized intense affections, with a high risk of becoming a traumatic experience. In this context, the support, through video calls, for PwD and their families, to minimize the impact of interruption of treatment presented itself as a viable alternative. The VDC program was built based on the demands, limitations, and possibilities of its users. The adhesion of PwD and caregivers and their adaptation to the use of technology were the main indicators of the feasibility of the VDC.

Social isolation is a risk factor for dementia8 and represents a risk of increased morbidity and mortality for the elderly in general and for the PwD and their caregivers. The lack of stimuli and the cognitive and physical inactivity resulting from social isolation can contribute to the appearance of neuropsychiatric symptoms, aggravate the clinical condition of PwD, and can increase the burden on caregivers8. In contrast, the literature reveals that the offer of physical, cognitive, and social stimuli positively impacts the well-being, functionality, and cognitive readiness of individuals.^16,23,24,25^ At the beginning of the pandemic, an Inter-Agency Standing Committee (IASC)^2^ highlighted the importance of interventions that favor the organization of routines and the adoption of creative mediation strategies, i.e., self-care practices and stress mediators, as strategies to reduce the traumatic impact of the pandemic.^2,3,4^

In this scenario, the possibility of a regular virtual intervention for PwD seemed impossible, considering that many healthy elderly people have resistance and difficulties in assimilation and adaptation to new languages and technologies^26^ and, as an aggravating factor, the limitations such as sensorial, cognitive impairment, and the need for adherence of caregivers in the face of functional dependence. Surprisingly, given the adherence and adaptation to the observed virtual modality, the use of the Internet proved to be a resource for social interaction.^27,28,29^

Therefore, it was necessary to adapt activities and methodologies to the virtual modality to a diversified program that met the needs. In general, even in face-to-face activities, flexibility and adaptation of activities and methodologies are necessary according to the characteristics, motivations, resources, and limitations of group members.^19,20,22,23^

Another aspect considered was the risk that virtual activities would represent an additional burden for caregivers. It is known that DC has as one of its objectives to offer a break for caregivers and reduce their burden and exposure to primary stressors,^16,19,23,24,25,30,31^ an objective made impossible by the pandemic. Given the complexity of the situation experienced by caregivers, the support in the pandemic had a triple function: psychoeducational to stimulate the adoption of adapted behavioral and biosafety strategies, minimizing isolation and its potential damage to the health of the dyad and the continuity of treatment. Therefore, the development of the VDC was made progressively in compliance with the guiding recommendations^2,3,4^ of psychosocial intervention during the pandemic and the demands presented by the caregivers. Most caregivers and PwD voluntarily adhered to the program and maintained their regular attendance for 12 months. Only three caregivers were unable to reconcile VDC activities with professional and family demands. The observed adherence suggests that the VDC played a role in the organization of its routines.

Regarding sustaining social engagement as fundamental to the mental health of PwD and their caregivers, Pearlin et al.^13,14^ emphasized the role of the social support offered by the caregiver’s relationship network, including the family and the assistance offered by the community-based health technologies, such as DCs, in managing stress, in linking the caregiver to a larger community, minimizing the isolation and alienation that many caregivers experience. Thus, the VDC seemed to favor the engagement of new members of the family network in care and the maintenance of the caregiver’s bond with their peers and with the team, minimizing their socioemotional isolation.

### Limitations

Old equipment and the instability of the Internet caused possible interruptions in the calls. The qualitative design included a small sample, an aspect that would not permit generalization. Also, we did not apply measures of cognition and neuropsychiatric symptoms, which would allow the effectiveness of the intervention.

The integrated care program, in the form of VDC, for PwD and their caregivers represented a challenge in which it was necessary to reinvent and make more flexible techniques, activities, and management, exercising creativity without losing the theoretical and ethical north of the work that the DC proposes, which is based on the sensitive encounter between science and compassion.

The knowledge produced in this experience and the results achieved can contribute to the expansion of this assistance to individuals and families who do not have access to face-to-face treatment. Thus, both in the present and in the future, remote assistance can be an excellent alternative to overcome barriers to access health devices.

## References

[1] Molina R (2006). Anais do 1º Seminário Nacional de Psicologia das Emergências e dos Desastres: contribuições para a construção de comunidades mais seguras.

[2] Inter-Agency Standing Committee (IASC) – Guia preliminar: Como lidar com os aspectos psicossociais e de saúde mental referentes ao surto de COVID-19. Versão 1.5 https://interagencystandingcommittee.org/system/files/2020-03/IASC%20Interim%20Briefing%20Note%20on%20COVID-19%20Outbreak%20Readiness%20and%20Response%20Operations%20-%20MHPSS%20%28Portuguese%29.pdf.

[3] Fundação Osvaldo Cruz (2020). Saúde Mental e Atenção Psicossocial na Pandemia COVID-19: Recomendações aos trabalhadores e cuidadores de idosos.

[4] Fundação Osvaldo Cruz (2020). Saúde Mental e Atenção Psicossocial na Pandemia COVID-19: Recomendações Gerais.

[5] Atkins JL, Masoli JA, Delgado J., Pilling LC, Kuo CL, Kuchel GA (2020). Preexisting comorbidities predicting severe Covid-19 in older adults in the UK biobank community cohort. J Gerontol A Biol Sci Med Sci..

[6] Kuo CL, Pilling LC, Atkins JL, Masoli JA, Delgado J, Kuchel GA (2020). APOE e4 Genotype Predicts Severe COVID-19 in the UK Biobank Community Cohort. J Gerontol A Biol Sci Med Sci..

[7] Dourado MC, Belfort T, Monteiro A, Lucena AT, Lacerda IB, Gaigher J (2020). COVID-19: challenges for dementia care and research. Dement Neuropsychol..

[8] Livingston G, Huntley J, Sommerlad A, Ames D, Ballard C, Banerjee S (2020). Dementia prevention, intervention, and care: 2020 report of the Lancet Commission. Lancet.

[9] Wilson RS, Evans DA, Bienias JL, Leon M, Schneider JA, Bennett DA (2003). Proneness to psychological distress is associated with risk of Alzheimer’s disease. Neurology..

[10] Wilson RS, Krueger KR, Arnold SE, Schneider JA, Kelly JF, Barnes LL (2007). Loneliness and risk of Alzheimer disease. Arch Gen Psychiatry..

[11] Kuiper JS, Zuidersma M, Voshaar RC, Zuidema AU, van den Heuvel ER, Stolk RP (2015). Social relationships and risk of dementia: A systematic review and meta-analysis of longitudinal cohort studies. Ageing Res Rev..

[12] Bzdok D, Dunbar RI (2020). The neurobiology of social distance. Trends Cogn Sci..

[13] Pearlin LI, Menaghan EG, Lieberman MA, Mullan JT (1981). The stress process. J Health Soc Behav..

[14] Pearlin LI, Mullan JT, Semple SJ, Skaff MM (1990). Caregiving and the stress process: an overview of concepts and their measures. Gerontologist..

[15] Chiao CY, Wu HS, Hsiao CY (2015). Caregiver burden for informal caregivers of patients with dementia: A systematic review. Int Nurs Rev..

[16] Maffioletti VL, Baptista MA, Santos RL, Rodrigues VM, Dourado MC (2019). Effectiveness of day care in supporting family caregivers of people with dementia: a systematic review. Dement Neuropsychol.

[17] Hughes CP, Berg L, Danziger WL, Coben LA, Martin RL (1982). A new clinical scale for the staging of dementia. Br J Psychiatry..

[18] Bertolucci PH, Brucki S, Campacci SR, Juliano Y (1994). O Mini-Exame do Estado Mental em uma População Geral: Impacto da Escolaridade. Arq Neuro-Psiquiatr..

[19] Maffioletti VL, Nigri FN, Baptista MA, Lima CA, Cavalcanti MT (2015). Centro dia para idosos com demência: um relato de experiência. Psicogeriatría..

[20] Feuerstein R, Feuerstein RS, Falik LH (2014). Além da inteligência: aprendizagem mediada e a capacidade de mudança do cérebro.

[21] Souza AM, Depresbiteris L, Machado OT (2003). A mediação como princípio educacional: bases teóricas das abordagens de Reuven Feuerstein.

[22] Da Ros SZ (2002). Pedagogia e Mediação em Reuven Feuerstein: o processo de mudança em adultos com história de Deficiência.

[23] Clare L (2017). Rehabilitation for people living with dementia: A practical frame-work of positive support. PLoS Med..

[24] Woods B, Aguirre E, Spector AE, Orrell M (2012). Cognitive stimulation to improve cognitive functioning in people with dementia. Cochrane Database Syst Rev.

[25] Mossello E, Caleri V, Razzi E, Di Bari M, Cantini C, Tonon E (2008). Day Care for older dementia patients: favorable effects on behavioral and psychological symptoms and caregiver stress. Int J Geriatr Psychiatry.

[26] Loreto ES, Ferreira GM (2014). Desafios e possibilidades para a Inclusão Digital da Terceira Idade. Rev Eletrônica Educ..

[27] Kachar V (2003). Terceira idade & informática: aprender revelando potencialidades.

[28] Wagner N, Hassanein K, Head M (2010). Computer use by older adults: A multi-disciplinary review. Comput Hum Behav..

[29] Liu Y, Tamura R (2020). In: Proceedings of the 8th International Conference on Kansei Engineering and Emotion Research..

[30] Tretteteig S, Vatne S, Rokstad AM (2017). The influence of day care centres designed for people with dementia on family caregivers - a qualitative study. BMC Geriatrics..

[31] Rokstad M, McCabe L, Robertson JM, StrandenÃ¦s MG, Tretteteig S, Vatne S (2019). Day care for people with dementia: A qualitative study comparing experiences from Norway and Scotland. Dementia (London).

